# Taking a chance: How likely am I to receive my preferred treatment in a clinical trial?

**DOI:** 10.1177/09622802221146305

**Published:** 2023-01-10

**Authors:** Stephen D Walter, Ondrej Blaha, Denise Esserman

**Affiliations:** 1 Department of Health Research Methodology, Evidence, and Impact, 3710 McMaster University, Hamilton, Ontario, Canada; 2 Department of Biostatistics, 50296 Yale School of Public Health, New Haven, CT, USA

**Keywords:** Clinical trials, treatment preference, randomisation, study design, patient recruitment

## Abstract

Researchers should ideally conduct clinical trials under a presumption of clinical equipoise, but in fact trial patients will often prefer one or other of the treatments being compared. Receiving an unblinded preferred treatment may affect the study outcome, possibly beneficially, but receiving a non-preferred treatment may induce ‘reluctant acquiescence’, and poorer outcomes. Even in blinded trials, patients’ primary motivation to enrol may be the chance of potentially receiving a desirable experimental treatment, which is otherwise unavailable. Study designs with a higher probability of receiving a preferred treatment (denoted as ‘concordance’) will be attractive to potential participants, and investigators, because they may improve recruitment and hence enhance study efficiency. Therefore, it is useful to consider the concordance rates associated with various study designs. We consider this question with a focus on comparing the standard, randomised, two-arm, parallel group design with the two-stage randomised patient preference design and Zelen designs; we also mention the fully randomised and partially randomised patient preference designs. For each of these designs, we evaluate the concordance rate as a function of the proportions randomised to the alternative treatments, the distribution of preferences over treatments, and (for the Zelen designs) the proportion of patients who consent to receive their assigned treatment. We also examine the *equity* of each design, which we define as the similarity between the concordance rates for participants with different treatment preferences. Finally, we contrast each of the alternative designs with the standard design in terms of gain in concordance and change in equity.

## 1 Introduction

Although patients and investigators in a clinical trial are often imagined to be in a state of equipoise between the merits of two competing treatments, in practice many of them will actually prefer one particular treatment. If patients are unblinded, and if they actually receive their preferred treatment, there may be an effect (possibly beneficial) on the study outcome. In contrast, patients who are not assigned to their preferred treatment may feel ‘reluctant acquiescence’ and resentment, which may lead to less favourable outcomes.^1–3^ Further, there is evidence that when patients receive their preferred treatment, the rates at which they withdraw from the trial are reduced, their adherence to the prescribed treatment increases, and outcomes are improved.^4^

Even when patients are blinded to treatments, they may decide to enrol in a trial just to have a chance of receiving a desirable experimental treatment, particularly if that treatment is not otherwise available outside the trial. In general, we speculate that trials which provide participants with a greater chance of receiving their desired treatment will be more attractive to them, and this may in turn improve patient recruitment and retention. Accordingly, we find it useful to consider the proportion of study participants who do indeed receive their preferred treatment when using various study designs.

In this article, the main focus is to evaluate the probability that a patient receives their preferred treatment, under several alternative trial designs where patient preferences may or may not be recognised and taken into account in the analysis. This probability will be referred to as the *concordance* rate. We compare concordance in the standard parallel group randomised trial design with each of the following: the two-stage randomised patient preference design; the fully randomised and the partially randomised patient preference designs; and the Zelen single and double consent designs.^5–7^ In addition to the study design itself, the concordance rate depends on the proportions of patients randomised to each of the treatments in question, on the distribution of patient preferences for those treatments, and in the case of the Zelen designs, on the proportion of patients who consent to randomisation. We describe the identifiability of the treatment, selection, and preference in the Zelen designs and compare them to earlier results on these features in the other designs.

We calculate the probability of concordance over all study participants, and within preference-specific subgroups of patients. We evaluate the *equity* of each design, which we define as the similarity between the concordance rates for participants with different treatment preferences. Parameter settings under which each study design has its maximum overall concordance or optimal equity are identified. In more generality, we explore and compare the overall concordance rates for various parameter configurations, and compare the improvements in equity that can be achieved over the standard design when a preference-based design is adopted. An R Shiny application has also been developed so that investigators can explore alternative design options for their own studies.

## 2 Methods

Suppose that two treatments, A and B, are to be compared and that the proportions of patients who prefer A or B are *α* and *β*, respectively. We will allow for the possibility that some proportion *γ* of patients are undecided (or indifferent) between treatments A and B. Hence *α* + *β* + *γ* = 1. We define patients who are assigned to their preferred treatment as being *concordant*. The group of concordant patients includes a mixture of those who prefer and are assigned to receive A, and those who prefer and are assigned to receive B; additionally, we regard indifferent patients as being satisfied with their assigned treatment (whichever it is), and hence they also constitute part of the concordant group.

**Figure 1.**
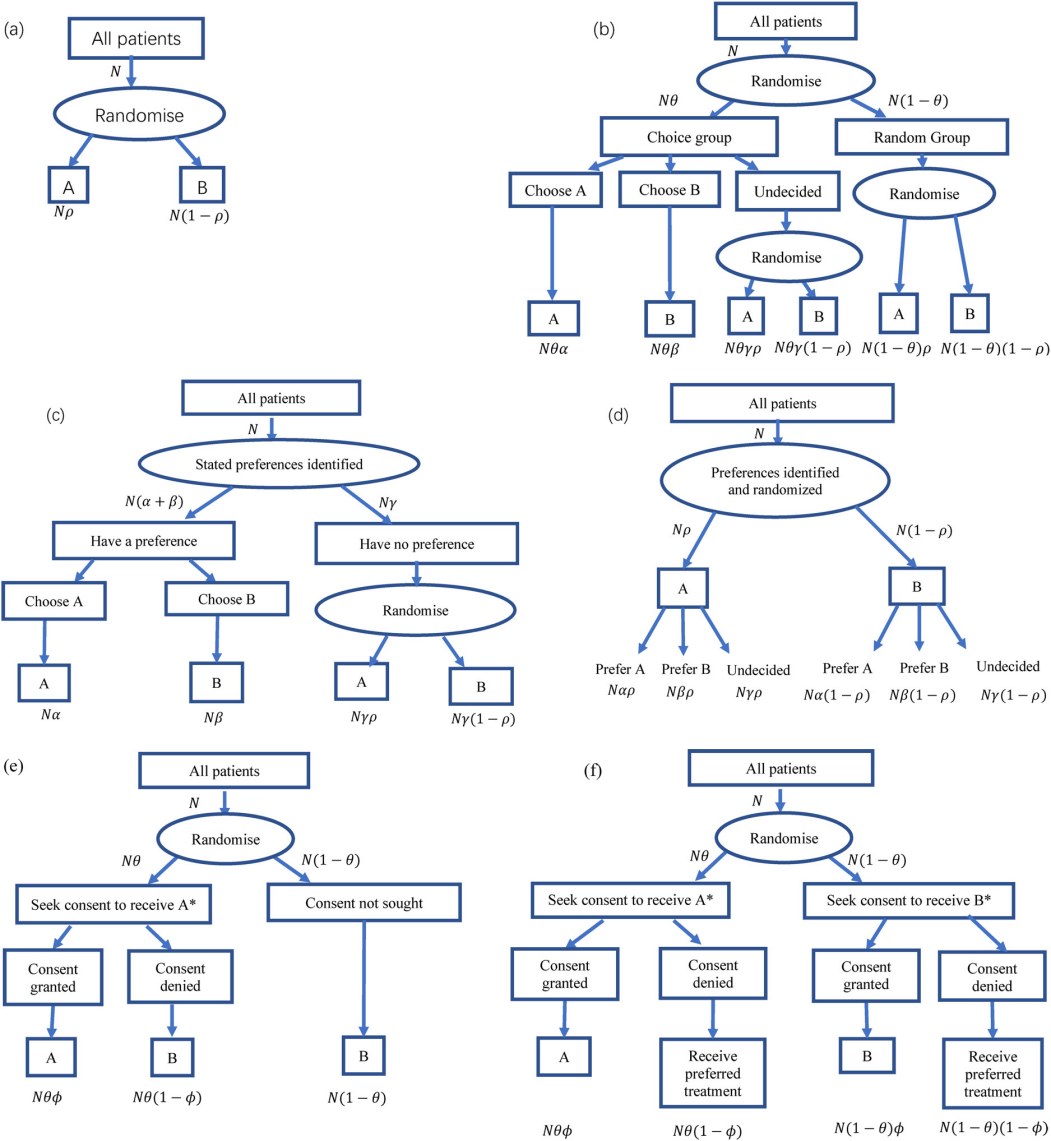
Six randomised trial designs: (a) conventional parallel group design; (b) two-stage randomised design; (c) partially randomised design; (d) fully randomised design; (e) Zelen single consent design; (f) Zelen double consent design. *In designs (e) and (f) details of treatment may alternatively be concealed or revealed at stage of seeking consent.

A variety of clinical trial designs are available, which may or may not take into account patient preferences. Some of the commonly used designs that we considered for our comparison are summarised in Table 1 and Figure 1. They are characterised by when or if preferences are identified, and which patients are either randomised or allowed to have their preferred treatment. In the standard parallel group design, all treatments are assigned at random, and patient preferences are not considered. In the two-stage design,^8–11^ a fraction *θ* of participants are randomised to a choice arm; within that arm, participants who have a definite choice of treatment are allowed to select it, while any undecided participants are assigned a treatment at random; the remaining fraction (1 − *θ*) are randomised in the usual way, as in a standard trial. In the fully randomised patient preference design, preferences are identified for all participants, but nevertheless, they are all randomised. In the partially randomised patient preference design, only the indifferent participants are randomised, while participants with a definite treatment preference are allowed to choose it.^1^

**Table 1.** Features of alternative trial designs.

	Design					
	Parallel group	Two-stage	Fully randomised	Partially randomised	Zelen: Single consent	Zelen: Double consent
Treatment preferences identified?	No	For a random subset of patients (In choice arm)	For all patients	For all patients	Not directly	Not directly
Which patients are allowed to choose treatment?	None	In choice arm	None	Patients with a stated preference	Patients in the single consent arm can agree to randomised treatment A or not	Patients in both consent arms can agree to randomised treatment (A or B) or not
Is treatment effect estimable?	Yes	Yes	Yes, overall and within preference subgroups	Yes, within preference subgroups	Yes. Unbiased if treatments are concealed, and consent is independent of patient preferences; biased if treatments are revealed	Yes. Unbiased if treatments are concealed, and consent is independent of patient preferences; biased if treatments are revealed
Selection effect estimable?	No	Yes	Yes, potentially biased	Yes, potentially biased	No	Yes if treatments concealed
Preference effect estimable?	No	Yes	Yes	No	No	Yes if treatments concealed

The Zelen design has several variants, which have been employed in several different situations.^5–7^ In the single consent version as we consider it, patients are randomised to (i) an arm in which an offer is made to receive an experimental treatment A if consent is granted, or treatment B (typically control or standard care) if consent is denied; or (ii) a control arm in which all patients receive treatment B. In the double consent method, patients in the first arm are asked for consent to receive A (as before), while in the second arm they are now asked for consent to receive B. If consent is denied in either arm, patients are then offered their preferred treatment. We will assume that patients who are indifferent or undecided about their preferred treatment at that stage would be randomised, in either arm. In each of the single and double consent approaches, investigators may conceal or reveal the details of both treatments at the point of asking for consent to accept the randomised treatment assignment. In the single consent version, patients in the control arm may be unaware that they are involved in a study to evaluate treatments and unaware of the fact that there are patients in another arm who are being offered the experimental treatment; this has led to certain ethical concerns and led to the development of the alternative double consent design.

We have previously reviewed^1,10^ each of these designs (except the Zelen designs) with respect to their estimability of the treatment effect, selection effects (which reflect different expected outcomes in patients who would prefer treatments A or B, if they were allowed to choose), and preference effects (which can be characterised as the interaction between the preferred and actual treatments). As seen in Table 1, the parallel group, two-stage, fully randomised and partially randomised designs all provide an estimate of the treatment effect, but with some caveats. The standard parallel group design permits unbiased estimation of the treatment effect but provides no information on preference or selection effects. The two-stage design has the attractive feature that it yields unbiased estimates of all three effects. The fully randomised design can validly estimate the treatment effect within preference-specific subgroups of patients, or the preference information can be discarded, and an unbiased estimate can be obtained, equivalent to that in the standard design. The treatment effect can be estimated with the partially randomised design within patient subgroups defined by their preferences; however, these are potentially biased estimates of the overall treatment effect.

Using an approach similar to that used for the other designs,^1,10^ we can similarly examine the estimability of effects in the Zelen designs; supporting derivations are shown in Supplemental Appendix 1. To summarise these results: if treatment identities are revealed at the stage of gaining patient consent in either of the single or double consent Zelen designs, the estimated treatment effect will then be potentially biased by preference and selection effects; the latter two effects are not identifiable. On the other hand, if treatment identities are concealed at that time, unbiased estimation of the treatment effect is possible with either the single or double consent designs, if the probability of a patient consenting to a given treatment is independent of any underlying treatment preference. Under the same assumption, the double consent, treatments-concealed design can then be analysed in the same way as a two-stage design, to yield valid estimates of the preference and selection effects.^1,10^ The plausibility of this key assumption is discussed later.

Here, our main objective is to compare concordance rates and equity values for the various trial designs. We also identify the scenarios in which concordance can be maximised over the possible distributions of treatment preferences among trial patients. Similarly, we investigate the cases when patient equity is optimised. For each design, we specify that whenever randomisation to treatments is used, it assigns fractions *ρ* and (1 − *ρ*) to treatments A and B, respectively; potential advantages of unequal randomisation have been discussed elsewhere.^9–14^

### 2.1 Standard parallel group design

Participants who prefer treatment A or B have probabilities *ρ* and (1 − *ρ*), respectively, of being assigned their preferred treatment, thus becoming concordant. The remaining participants are indifferent. Hence, the concordance rates for these subgroups and over all patients in the study are given by

(1) Pr(concordant|preferA)=ρ

(2) Pr(concordant|preferB)=1−ρ

(3) Pr(concordant|indifferent)=1

(4) Pr(concordant)=αρ+β(1−ρ)+γ=1−α(1−ρ)−βρ

The overall proportion of concordant patients in (4) is obtained by weighting the preference-specific concordance rates by the components (*α, β*, *γ*) of the treatment preference distribution. Several implications are evident. First, the concordance among A-preferers increases as the rate of assignment to treatment A (*ρ*) increases, and vice versa for treatment B. Second, if treatments are equally preferred (*α* = *β*), the overall concordance rate (denoted by C) simplifies to (1 + *γ*)/2, which achieves its maximum value of C = 1 whenever *γ* = 1; this is a degenerate case when no patients have a treatment preference. Third, there are two additional degenerate cases when maximum concordance occurs: when *ρ* = 1 and *α* = 1 (or when *ρ* = 0 and *β* = 1), so everyone is randomised to A (or B) and all patients prefer it. These two cases present scenarios where there is only one treatment group, and therefore no comparison of treatments is possible; hence they are of no practical interest. Fourth, while the previous degenerate cases are not conditional on the values of any parameters, it is also worthwhile to examine concordance as a function of the definitive preference rates *α* and *β*, while conditioning on *γ* and *ρ* as fixed parameters. Recalling that *β* = 1 − *α* − *γ*, and differentiating (4) with respect to *α*, we can identify the scenarios for *conditional maximum concordance* over the possible distributions of treatment preference (while disregarding indifferent patients, and with a given randomisation ratio). The maximum possible overall concordance C is again (1 + *γ*)/2, which occurs in this setting when *ρ* = 1/2, independent of the preference rates *α* and *β*.

Finally, an alternative is to maximise concordance over the randomisation parameter *ρ*, which is under the control of the investigators. If we differentiate (4) with respect to *ρ*, we find that maximum concordance is achieved if *α* = *β*, and in that case, the maximum concordance itself is 1 − *α*. Here, the maximum concordance is not a function of *ρ*, and so the value of *ρ* should be chosen based on considerations other than the preference distribution.

We define *equity* as the difference in concordance rates between A- versus B-preferers. Indifferent participants are always concordant, so they are not relevant for comparisons of equity values, either within or between designs. The ideal equity value is zero, when the design equally favours A- and B-preferers; positive equity values imply greater concordance in favour of A-preferers, while negative equity implies that B-preferers are favoured. From (1) and (2)

(5) Equity=ρ−(1−ρ)=2ρ−1

Hence, the ideal equity value is achieved when *ρ* = 1/2, this is when equal numbers of participants are randomised to A and B. Note that this occurs independently of the preference distribution. If *ρ* > 1/2, the resultant positive equity value favours A-preferers, but if *ρ* < 1/2, the negative equity favours B-preferers. Table 2 summarises concordance rates and equity for this and the other study designs which follow; it also describes the scenarios where concordance is maximised as a function of the distribution of patient preferences, conditionally on the other parameters. Table 3 is similar but covers maximisation of concordance over the design parameters which are under the control of investigators, again conditionally on the remaining parameters.

**Table 2.** Concordance rates and equity values in alternative trial designs.

	Design						
	Parallel group and fully randomised	Two-stage	Partially randomised	Zelen single consent, treatments concealed	Zelen single consent, treatments revealed	Zelen double consent, treatments concealed	Zelen double consent, treatments revealed
Concordance rate for patients who prefer treatment A	*ρ*	*θ* + (1 − *θ*)*ρ*	1	*θφ*	*θ*	1 − *φ*(1 − *θ*)	1
Concordance rate for patients who prefer treatment B	1 − *ρ*	*θ* + (1 − *θ*)(1 − *ρ*)	1	1 − *θφ*	1	1 − *φθ*	1
Concordance rate	*αρ* + *β*(1 − *ρ*) + *γ*	*θ* + (1 − *θ*) [*αρ* + *β*(1 − *ρ*) + *γ*]	1	*αθφ* + *β*(1 − *θφ*) + *γ*	*αθ* + *β* + *γ*	1 − *φ*[*α*(1 − *θ*) + *βθ*]	1
Scenarios to maximise concordance (C = 1)	1) *γ* = 1^a^ 2) *α* = 1, *ρ* = 1^a^ 3) *β* = 1, *ρ* = 0^a^	1) *γ* = 1^a^ 2) *θ* = 1^a^ 3) *α* = *ρ* = 1^a^ 4) *β* = 1, *ρ* = 0^a^	Always	1) *γ* = 1^a^ 2) *α* = 1, *θ* = *φ* = 1^a^ 3) *β* = 1, *θ* = *φ* = 0^a^	1) *γ* = 1^a^ 2) *β* = 1^a^ 3) *θ* = 1^a^	*γ* = 1^a^ *φ* = 0^a^ *α* = *θ* = 0^a^ *β* = *θ* = 1^a^	Always
Scenarios where concordance is conditionally^b^ maximised	*ρ* = 1/2^(1)^	*ρ* = 1/2^(2)^	Always	*θφ* = 1/2^(3)^	*θ* = 1^a^^(4)^	*φ* = 0^a^^(5)^ *θ* = 1/2	Always
Conditional maximum concordance	(1 + *γ*)/2	*θ* + (1 − *θ*)(1 + *γ*)/2	1	(1 + *γ*)/2	1	1 (if *φ* = 0) 1 − 1/2 (1 − *γ*) (if *θ* = 1/2)	1
Equity (A vs. B)	2*ρ* − 1	(1 − *θ*)(2*ρ* − 1)	0	2*θφ* − 1	*θ* − 1	*φ*(2*θ* − 1)	0
Scenarios to optimise equity	*ρ* = 1/2	*ρ* = 1/2 *θ* = 1^a^	Always	*θφ* = 1/2	*θ* = 1^a^	*φ* = 0^a^ *θ* = 1/2	Always
Conditional optimal equity	0	0	0	0	0	0	0

*ρ* = proportion randomised to treatment A in standard design, and in a random arm of 2-stage design; (*α*, *β*) = preference rates for treatment A or B; *γ* = undecided rate; *θ* = proportion randomised to choice arm in two-stage design, or to A-consent arms in Zelen designs; *φ* = proportion of patients who consent to randomised treatment in Zelen concealed designs.

^a^ Implies degenerate design.

^b^ Maximised over definitive treatment preference rates (*α* and *β*), conditional on fixed parameters (1) *γ*, *ρ*; (2) *γ*, *ρ*, *θ*; (3) *γ*, *θφ*; (4) *γ*, *θ*; (5) *γ*, *φ*, *θ*.

**Table 3.** Concordance rates in alternative trial designs; maximised with respect to design parameters.

	Design						
	Parallel group and fully randomised	Two-stage	Partially randomised	Zelen single consent, treatments concealed	Zelen single consent, treatments revealed	Zelen double consent, treatments concealed	Zelen double consent, treatments revealed
Concordance rate	*αρ* + *β*(1 − *ρ*) + *γ*	*θ* + (1 − *θ*)[*αρ* + *β*(1 − *ρ*) + *γ*]	1	*αθφ* + *β*(1 − *θφ*) + *γ*	*αθ* + *β* + *γ*	1 − *φ*[*α*(1 − *θ*) + *βθ*]	1
Scenarios where concordance is conditionally^a^ maximised	*α* *=* *β*^(1)^	*α* *=* *β*^(2)^	Always	*α* = *β*^(3)^ *φ* = 0^b^	*α* = 0^(4)^	*φ* = 0^(5)^ *α* = *β*	Always
Conditional maximum concordance	*1* − *α*	1 − *α*(1 − *θ*)	1	*1* − *α*	1	1 (if *φ* = 0) 1 − *φα* (if *α* = *β*)	1

*ρ* = proportion randomised to treatment A in standard design, and in a random arm of 2-stage design; (*α*, *β*) = preference rates for treatment A or B; *γ* = undecided rate; *θ* = proportion randomised to choice arm in two-stage design, or to A-consent arms in Zelen designs; *φ* = proportion of patients who consent to their randomised treatment in Zelen concealed designs.

^a^ Maximised over *ρ* (parallel group, fully randomised, and two-stage design), or *θ* (Zelen designs), conditional on fixed parameters (1) *α*, *β*, *γ*; (2) *α*, *β*, *γ*, *θ*; (3) *α*, *β*, *γ*, *φ*; (4) *γ*, *θ*; (5) *α*, *β*, *φ*

^b^ Implies degenerate design.

### 2.2 Two-stage design

In the two-stage design, a fraction *θ* of the participants is randomly assigned to the choice arm. These participants are then given their preferred treatment (if they have one) and hence become concordant. The complementary fraction (1 − *θ*) of participants are assigned to the random arm and participants in this group are given treatment according to that randomisation. By virtue of the first stage randomisation, the participants in the random arm are a random sample of all the participants, and hence their expected concordance rates are the same as if they had been in a standard trial, that is, equations (1) to (4) apply once again to them. Undecided participants in the choice arm are randomised to treatments A and B at the same rates (*ρ* and 1 − *ρ*) as elsewhere.

Patients who prefer treatment A become concordant in one of two ways: either if they are randomly assigned to the choice arm or if they are assigned to the random arm and then randomised to A, events which occur with probabilities *θ* and (1 − *θ*) *ρ,* respectively. Similar logic applies to the patients who prefer B or who are indifferent. Hence, the concordance rates and equity for this design are as follows:

(6) Pr(concordant|preferA)=θ+(1−θ)ρ

(7) Pr(concordant|preferB)=θ+(1−θ)(1−ρ)

(8) Pr(concordant|indifferent)=1

(9) Pr(concordant)=θ+(1−θ)[αρ+β(1−ρ)+γ]=(1−θ)[(α−β)ρ−α]+1

(10) Equity=(1−θ)(2ρ−1)

Inspecting (9), we see that there are four scenarios, all degenerate, where the unconditional maximum concordance rate of C = 1 can be attained. First, if all patients are indifferent (*γ* = 1), and hence they are all concordant; in this case, the design devolves to a standard parallel group study, with no definite treatment choices occurring. Second, if *θ* *=* 1, that is, all patients are given a choice of treatments and no randomised treatment comparison is possible. Third, if *α* = 1 and *ρ* = 1, all patients prefer A and the random treatment assignments are all to A; hence no patients receive B and no treatment comparison is possible. Fourth, the complementary scenario arises if *β* *=* 1 and *ρ* = 0, and all patients receive B. Additionally, if we assume that *γ*, *ρ*, and *θ* are all fixed, and we differentiate (9) with respect to *α*, the conditional maximum overall concordance is then *θ* + (1 − *θ*)(1 + *γ*)/2, which occurs when *ρ* = 1/2, regardless of the relative preference rates *α* and *β* for treatment. Finally, if we relax the conditioning on *ρ*, but keep *θ* as fixed, the conditional maximum concordance then occurs when *α* = *β*, that is, when the treatments are equally preferred among patients with a definitive preference, for any value of the treatment randomisation parameter *ρ*.

For any value of *θ*, the ideal equity of 0 is achieved when *ρ* = 1/2, regardless of the treatment preference rates. Again, patients who prefer treatment A are favoured if investigators decide to use *ρ* > 1/2. Equity can also attain its ideal value in the degenerate case when *θ* = 1, that is, all patients are assigned to the choice arm, but this is then at the expense of losing a randomised comparison of treatments.

### 2.3 Comparison of standard and two-stage designs

We may now consider the gain in concordance rates and the change in equity if a two-stage design is adopted instead of a standard design. We obtain:

(11) Gaininconcordance|preferA=θ(1−ρ)

(12) Gaininconcordance|preferB=θρ

(13) Gaininconcordance|indifferent=0

(14) Overallgaininconcordance=θ[1−αρ−β(1−ρ)−γ]=θ[α(1−ρ)+βρ]

(15) Changeinequity(Avs.B)=θ(1−2ρ)

From the positive values in (11) and (12), we see that concordance is necessarily higher among patients with a definite preference for either treatment when using the two-stage design (except for the degenerate scenario when no participants are allowed into a choice arm (*θ* = 0) in which case the two designs are equivalent). Intuitively, this is because of the existence of the choice arm, within which all participants become concordant. Because the preference subgroups both then have improved concordance, the overall concordance rate is also inherently increased. Gains in the concordance rate and the changes in equity for this and all the other study designs considered throughout this article are summarised in Table 4.

**Table 4.** Gain in concordance and change in equity for alternative designs when compared to the standard, parallel group design.

	Two-stage	Zelen single consent, treatments concealed	Zelen single consent, treatments revealed	Zelen double consent, treatments concealed	Zelen double consent, treatments revealed
Gain in Concordance rate for patients who prefer treatment A	*θ*(1 − *ρ*)	*θφ* − *ρ*	*θ* − *ρ*	1 − *φ*(1 − *θ*) − *ρ*	1 − *ρ*
Gain in concordance rate for patients who prefer treatment B	*θρ*	*ρ* − *θφ*	*ρ*	*ρ* − *θφ*	*ρ*
Gain in overall concordance rate	*θ*[1 − *αρ* − *β*(1 − *ρ*) − *γ*] = *θ* [*α* (1 − *ρ*) + *βρ*]	(*α* − *β*)(*θφ* − *ρ*)	*α*(*θ* − *ρ*) + *βρ*	(*α* − *β*)(*θφ* − *ρ*) + *α*(1 − *φ*)	*α*(1 − *ρ*) + *βρ*
Change in equity (A vs. B)	−*θ*(2*ρ* − 1)	2(*θφ* − *ρ*)	*θ* − 2*ρ*	*φ*(2*θ* − 1) − (2*ρ* − 1)	1 − 2*ρ*

*ρ* = proportion randomised to treatment A in standard design, and in a random arm of 2-stage design; (*α*, *β*) = preference rates for treatment A or B; *γ* = undecided rate; *θ* = proportion randomised to choice arm in two-stage design, or to A-consent arms in Zelen designs; *φ* = proportion of patients who consent to randomised treatment in Zelen concealed designs.

Results (5) and (10) show that equity is positive in both designs (favouring A-preferers) if more patients are randomised to A (i.e. *ρ* > 1/2), and that the two-stage design necessarily possesses equity closer to the ideal zero value, by a factor (1 − *θ*). Additionally, these results indicate that equity is always closer to the ideal zero with the two-stage design compared to the standard design for any value of *ρ*, in proportion to the number of participants allocated to the choice arm. When *ρ* = 1/2, both designs have ideal equity, as noted earlier.

### 2.4 Fully randomised and partially randomised patient preference designs

In the fully randomised patient preference design, whilst treatment preferences are elicited for all participants, subjects are all randomised anyway, so there is no impact of preferences on concordance. Therefore, the concordance rates in the fully randomised preference design are the same as in the standard design.

In the partially randomised patient preference design, patients with a treatment preference are allowed to receive it, and only the indifferent participants are randomised. Thus, all patients become concordant. Consequently, equity achieves its ideal value of zero regardless of the preference distribution.

### 2.5 Zelen designs

In the Zelen single consent design, patients are randomised with probability *θ* into a consent arm, this being the only arm where the experimental treatment A is potentially available. Details of the treatments under comparison may or may not be concealed at the stage of randomisation (cf. Figure 1(e)). If they are concealed, we assume that a proportion *φ* of patients in this arm accepts the randomisation, and thus receive treatment A. However, if the treatments’ details are revealed, patients can guarantee the receipt of their preferred treatment (if they have one). Patients who prefer B will receive it unless they are randomised to a consent arm with concealed treatments and accept randomisation. The resultant preference-specific and overall concordance rates along with the corresponding equity values for each of these scenarios are summarised in Tables 2 and 3. For example, a patient who prefers treatment A will receive it (and hence be concordant) if he/she is randomised to the consent arm, gives consent, and accepts the concealed treatment (A). This occurs with probability *θϕ*, assuming (as noted earlier) that granting consent is independent of treatment preference. Patients who prefer B will receive it if they are either randomised to the consent arm but refuse to consent or are randomised to the standard treatment B in the other arm; this happens with probability *θ* (1 − *ϕ*) + (1 − *θ*) = 1 − *θϕ*.

The logic for the Zelen single consent, treatments-revealed and double consent designs is similar.

### 2.6 Maximising concordance and optimising equity

Table 2 indicates the various scenarios (some of them being degenerate cases) where concordance is maximised or equity is optimised, either unconditionally, or over the treatment preference parameters *α* and *β*. Table 3 shows corresponding results when concordance is conditionally maximised over design parameters that are under the control or choice of the investigators. These parameters are the treatment randomisation rate *ρ* in the parallel group, fully randomised, and two-stage designs, or the proportion of patients assigned to a consent arm *θ* in the Zelen designs. Both tables indicate which of the remaining parameters are regarded as fixed when conditional maximisation is involved.

In some of these cases, comparing outcomes between treatments is impossible, for instance, if all patients are randomised to the same treatment. However, it is of interest to find scenarios for conditionally maximised concordance rates, where we regard some of the model parameters (such as the proportion *ρ* of patients randomised to treatment A or the proportion *γ* of patients who are undecided or indifferent about treatment preferences) as fixed, or where the maximisation is over the definitive preference rates *α* and *β*. As shown in Table 2, this leads to conditions such as equal randomisation to A and B in the standard trial (*ρ* = 1/2). These conditions may or may not lead to the overall concordance achieving its maximum value of 1, depending on other parameter settings. The partially randomised and Zelen's double consent, treatments-revealed designs do always achieve concordance rates of 1, but this is at the cost of potentially biased estimates or non-identifiability of the various effects in question.

When the maximisation is over the randomisation parameter *ρ* in the standard parallel group, fully randomised, or two-stage designs, maximum concordance occurs when *α* *=* *β* (Table 3). In general, that maximum is a function of *α*, with values <1. Perfect concordance with C = 1 can be achieved if *α* = *β* = 0, but that would entail a loss of estimability of the preference and selection effects in the two-stage design because all patients would be indifferent or undecided about their preferred treatment. In such a situation, those patients should be randomised for treatments, and the design then devolves into a standard parallel group design.

A further possibility occurs with maximisation over *θ* in either of the Zelen treatment concealed designs, when *ϕ* = 0, which means that no one gives consent to a randomised treatment assignment (Table 3); in the single consent concealed treatment Zelen design, this implies that no patients receive treatment A, whereas in the double consent concealed treatment design, all patients receive their preferred treatment. These scenarios again imply a loss of estimability of some of the effects of interest.

A similar approach is possible when considering equity. For instance, optimal equity can be achieved in the standard and two-stage designs by using equal randomisation rates to treatments A and B (*ρ* = 1/2), or in the Zelen single consent, treatments-concealed design by ensuring *θϕ* = 1/2. Perfect equity of zero is possible with each of the alternative designs, but again one needs to consider what can be estimated, while avoiding degenerate scenarios. For instance, in the Zelen single consent, treatments-revealed design, equity is optimised if all patients are randomised to the consent arm (*θ* = 1), but this makes the design degenerate and rules out unbiased estimation of any of the effects.

### 2.7 Comparison of standard and Zelen designs

As we have done previously with the two-stage design, we can compare the concordance rates and equity values of the Zelen designs against the standard parallel group design. For the *single consent, treatments-concealed design*, we have

Gain in concordance | prefer A = *θϕ* − *ρ*.
Gain in concordance | prefer B = *ρ* − *θϕ*.
Gain in concordance | indifferent = 0.
Overall gain in concordance = (*α* − *β*)(*θϕ* − *ρ*).
Change in equity (A vs. B) = 2 (*θϕ* − *ρ*).

Here, concordance gains for patients who prefer A are mirrored by concordance losses of the same amount for B-preferers and vice versa. Whether there is an overall gain in concordance depends on the treatment randomisation rate, the consent rate, and the relative number of patients who prefer treatment A over B. For instance, if more patients prefer A, then overall concordance improves if (a) more patients are randomised to the consent arm in the Zelen design, (b) more patients grant consent, or (c) fewer patients are randomised to A in the standard trial. Similarly, equity can become nearer or farther from the ideal value of zero, depending on these parameters.

For the *single consent, treatments-revealed design*, there is a degenerate case (*θ* = 1) when concordance is conditionally maximised. Equity is always imbalanced in favour of B-preferers, unless *θ* = 1, which is the same degenerate case. Comparing this design to the standard trial yields

Gain in concordance | prefer A = *θ* − *ρ.*
Gain in concordance | prefer B = *ρ.*
Gain in concordance | indifferent = 0.
Overall gain in concordance = *α* (*θ* − *ρ*) + *βρ.*
Change in equity (A vs. B) = *θ* − 2*ρ*.

Thus, under this design, concordance is always better for B-preferers, compared to the standard design.

With the *double consent, treatments-concealed design*, we again have several degenerate cases where concordance is maximised or equity is optimised. However, in this case, we have additional non-degenerate solutions if *θ* = 1/2. Comparing this design to the standard trial gives

Gain in concordance | prefer A = 1 − *ϕ* (1 − *θ*) − *ρ.*
Gain in concordance | prefer B = *ρ* − *θϕ*.
Gain in concordance | indifferent = 0.
Overall gain in concordance = (*α* − *β*)(*θϕ* − *ρ*) + *α* (1 − *ϕ*).
Change in equity (A vs. B) = *ϕ*(2*θ* − 1) − (2*ρ* − 1).

For this comparison, there is no clear winner based on overall concordance or equity.

Investigators should explore parameter settings of interest to aid in their choice of design. For this purpose, we have developed an interactive R Shiny^15^ web-based application that visually summarises all the presented results, including group-specific as well as overall concordance rates, equity, gain in concordance, and change in equity for all the considered designs over the standard design. This app can be found at: https://ondrej.shinyapps.io/Concordance/.

Finally, for the *double consent, treatments-revealed design*, all study participants are aware of the treatment they are being offered based on randomisation and hence anyone with a preference can receive their preferred treatment and is therefore concordant. As usual, indifferent or undecided patients are always concordant. Thus, concordance is universal, and equity is always perfect. Compared to the standard design, we have

Gain in concordance | prefer A = 1 − *ρ.*
Gain in concordance | prefer B = *ρ.*
Gain in concordance | indifferent = 0.
Overall gain in concordance = *α* (1 − *ρ*) + *βρ.*
Change in equity (A vs. B) = 1 − 2*ρ*.

With this Zelen design, both A- and B-preferers gain concordance. Equity is always ideal, and the standard design matches it only when *ρ* = 1/2.

## 3 Numerical results

We present some numerical comparisons of concordance and equity values between the two-stage and Zelen study designs versus the standard parallel group design. We do not discuss these quantities for the fully randomised patient preference design, because they are the same as in the standard design. We also omit mention of the partially randomised patient preference design because its concordance and equity profiles are relatively simple and straightforward (see Table 2).

### 3.1 Two-stage versus standard designs

**Figure 2.**
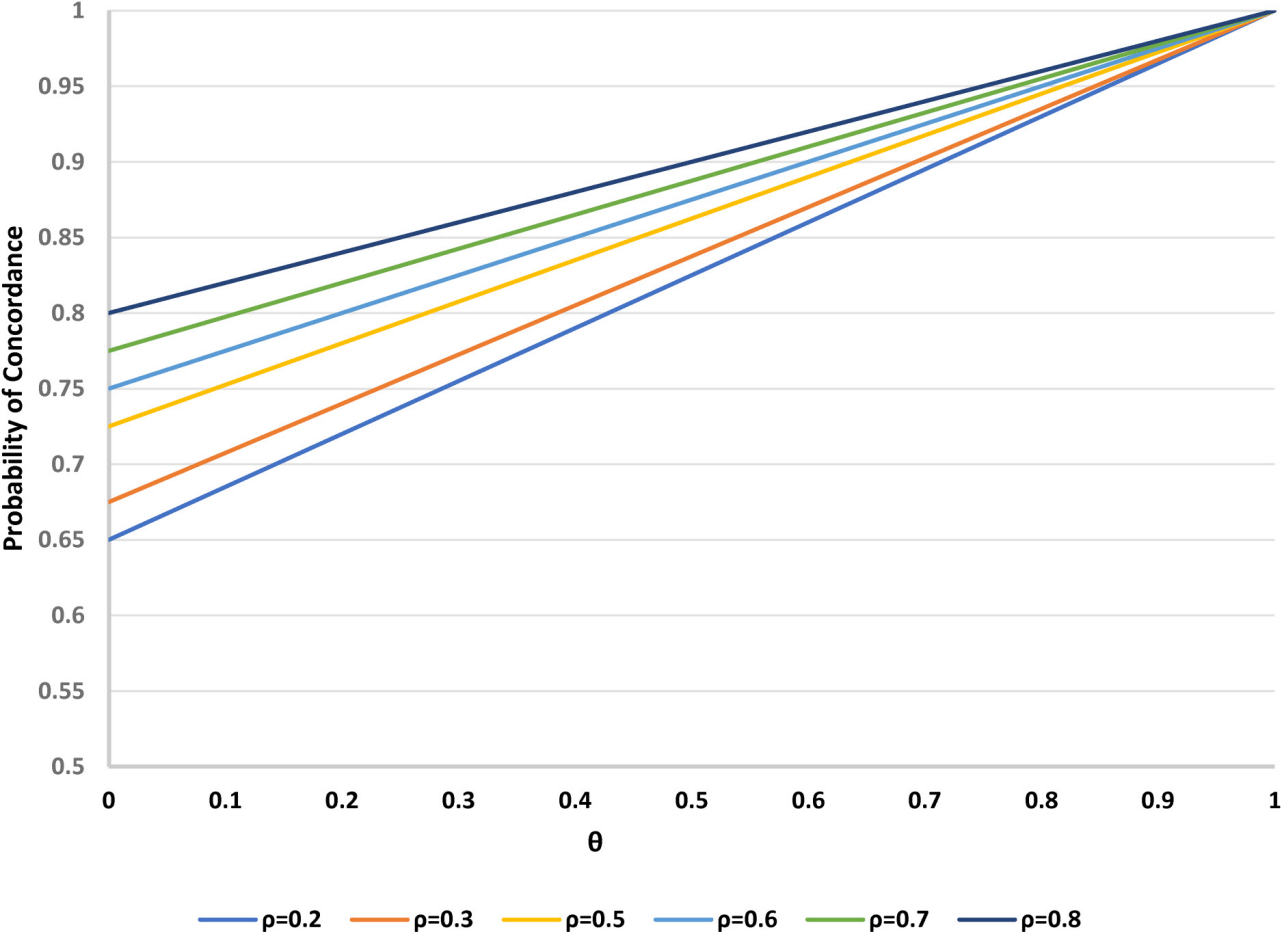
Probability of concordance versus *θ* (proportion randomised to the choice arm in a two-stage design), for varying *ρ* (proportion randomised to treatment A). Fixed parameters: *α* = 0.4 (proportion preferring treatment A); *β* = 0.15 (proportion preferring treatment B); *γ* = 0.45 (proportion undecided). *θ* = 0 corresponds to the standard design.

No matter the parameter space, there will always be a larger probability of concordance under the two-stage design relative to the standard parallel group clinical trial (which has *θ* = 0). If the remaining parameters (*α*, *β*, *γ*, *ρ*) are regarded as fixed, the overall probability of concordance increases with the proportion *θ* of participants assigned to the choice group in the two-stage design. Figure 2 shows this relationship for some typical values of the preference distribution (*α* = 0.4, *β* = 0.15, *γ* = 0.45) and for a range of possible values of the randomisation parameter *ρ*. As expected, concordance approaches 1 as *θ* approaches 1, when all patients can choose their treatment. For smaller values of *θ*, the linear relationship of the concordance rate to *ρ* is evident. Guidance is available elsewhere^6,8^ on how to determine the optimal allocation ratio *θ* for the two-stage design.

**Figure 3.**
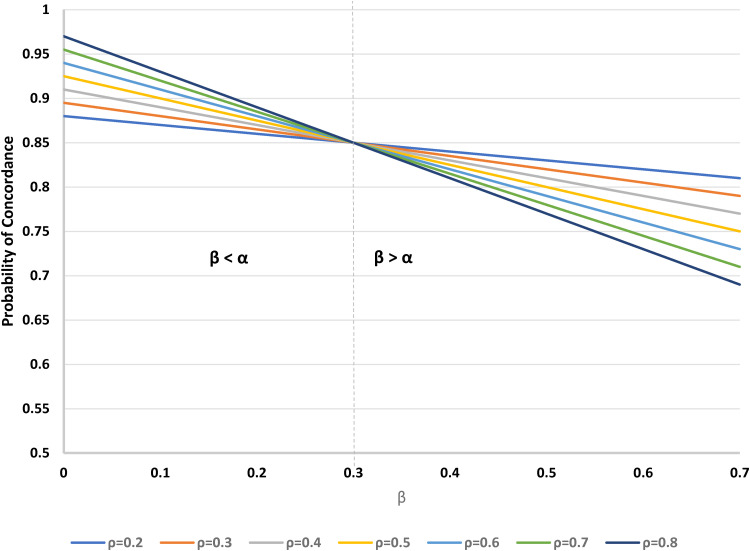
Probability of concordance versus *β* (proportion preferring treatment B), for varying *ρ* (proportion randomised to treatment A) in a two-stage design. Fixed parameters: *α* = 0.3 (proportion preferring treatment A); *θ* = 1/2 (proportion randomised to the choice arm).

For fixed *θ* and *α*, concordance decreases as *β* increases. More specifically, when *β* > *α*, as *ρ* increases, concordance decreases, but when *β* < *α*, concordance increases with *ρ*; when *β* = *α*, there is a crossover point, with the concordance rate being constant. See Figure 3 for a typical example, where *α* = 0.3 and *θ* = 1/2. Note that in this example, the relationships of concordance to *β* are negative, despite the apparent positive dependence implied by (9); this is because of the constraint that *α* + *β* + *γ* = 1; with *α* being fixed, as *β* increases, there is a corresponding reduction in *γ* (the proportion of undecided participants), and this latter group has 100% concordance, as opposed to the B-preferers, for whom there is less than certain concordance. Similar patterns occur if instead we take *β* as fixed and evaluate the impact of changes in *α*.

**Figure 4.**
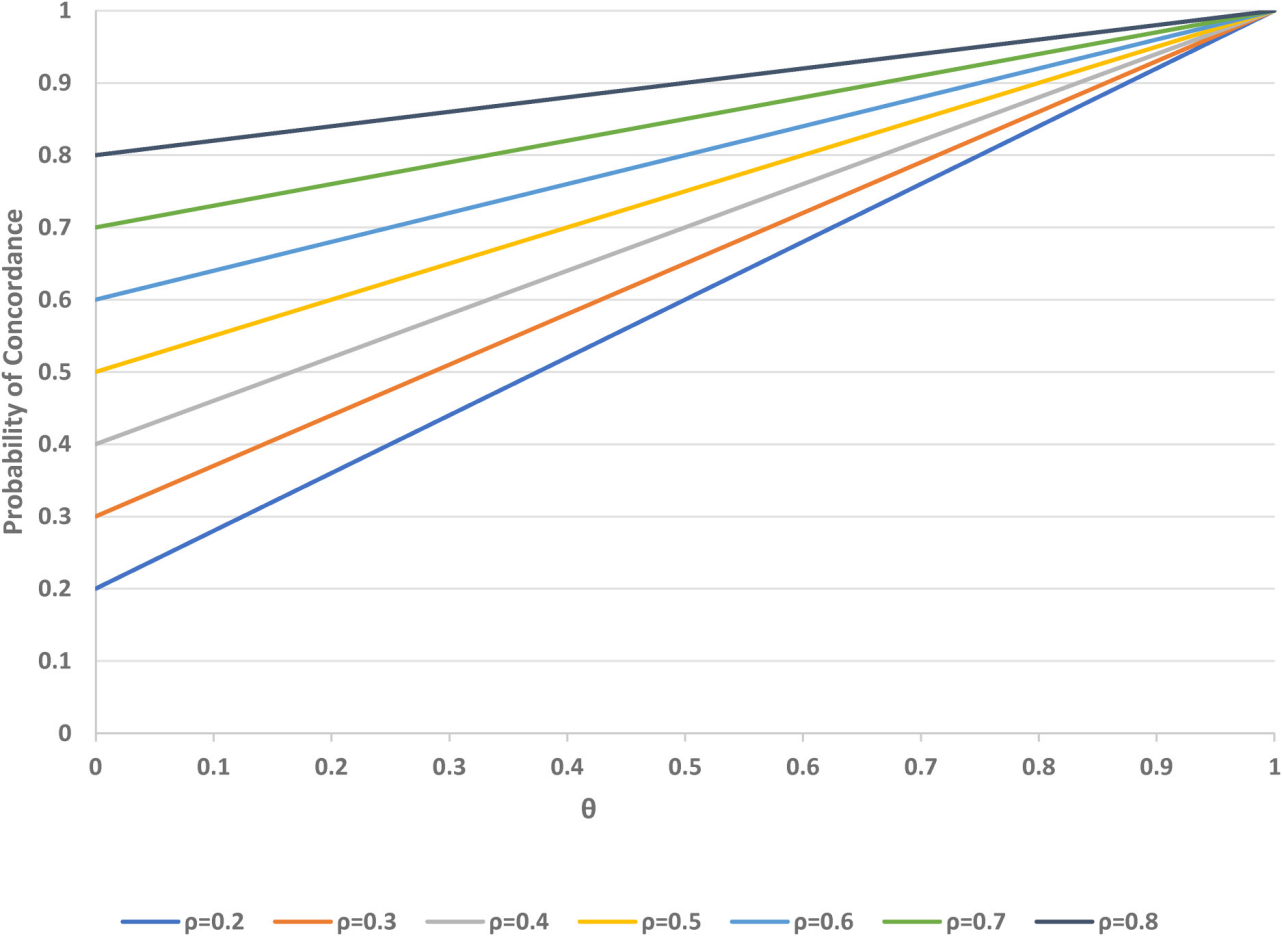
Probability of concordance for individuals who prefer treatment A, versus *θ* (proportion randomised to the choice arm) for varying *ρ* (proportion randomised to treatment A) in a two-stage design. *θ* = 0 corresponds to the standard design.

Next, we examine the probability of concordance among participants who prefer treatment A, as a function of *ρ* and *θ* (see Figure 4). As *θ* increases from 0 (as in a standard trial design) to 1 (a trial in which all participants can choose their treatment) the concordance rate increases linearly. The concordance also increases with *ρ*, the proportion randomised to A, and there is a symmetrically negative dependence on *ρ* for participants who prefer treatment B.

**Figure 5.**
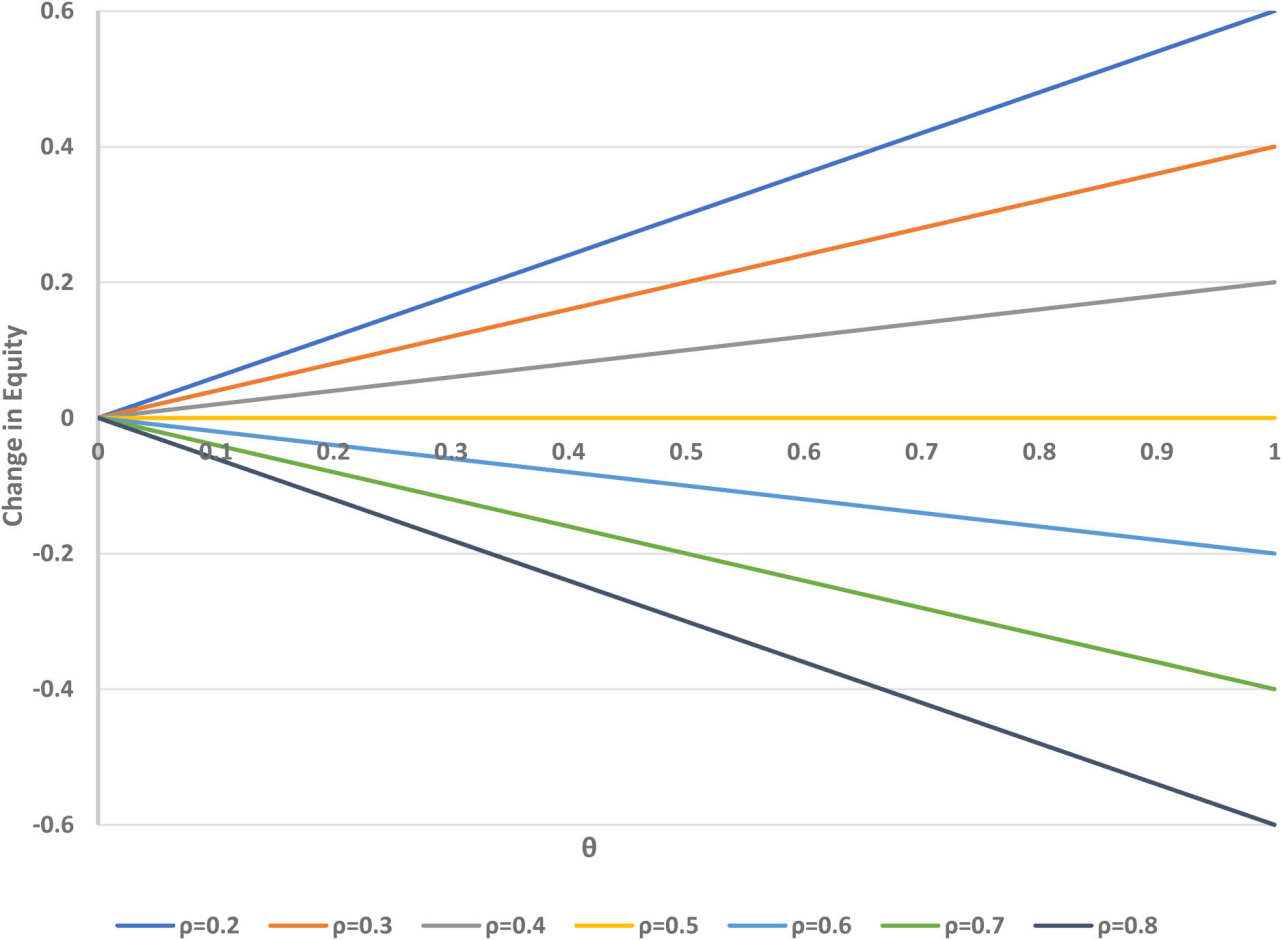
Change in equity versus *θ* (proportion randomised to the choice arm in a two-stage design) over varying *ρ* (proportion randomised to treatment A). *θ* = 0 corresponds to the standard design.

Figure 5 shows the potential change in equity (measured in terms of concordance for A-preferers minus concordance for B-preferers) associated with using the two-stage design, compared to the standard design, as a function of *ρ* and *θ*. If fewer people are randomised to A than B (i.e. *ρ* < 1/2), there is an increase in the equity value, but there is a decrease if *ρ* > 1/2, when more participants are randomised to B rather than A.

If *ρ* = 1/2, the standard (*θ* = 0) and two-stage designs (with any value of *θ*) have equal equity. The impact of the randomisation parameter *ρ* is greater for larger values of *θ*, when more participants are assigned to the choice group in the two-stage design. In a standard trial, no choice of treatment is allowed, and thus no change in equity is possible by that method. Figure 4 indicates that changes in *ρ* can improve or reduce the relative equity in the two-stage design for one or the other group of participants, but recall that equity in each design is closer to its ideal value of zero when *ρ* is close to 1/2.

The important results from the two-stage design can be further examined in the interactive online application available at https://ondrej.shinyapps.io/Concordance/. This application contains key functions (the probability of concordance – equation (9); the probability of concordance for patients who prefer treatments A and B – equations (6) and (7), respectively; equity – equation (10); overall gain in concordance – equation (14); gain in concordance for A- and B-preferers – equations (11) and (12); and the change in equity – equation (15) by using the two-stage design vs. the standard design) of all of the design parameters which are subject to change (i.e. *α*, *β*, *ρ*, and *θ*). This application is a useful tool for investigators weighing alternative trial designs, particularly as it affords them the opportunity to explore concordance and equity with the treatments under consideration. As stated earlier, this could improve the ability to recruit participants to clinical trials and can highlight the potential advantages of considering a two-stage design.

### 3.2 Zelen versus standard designs

**Figure 6.**
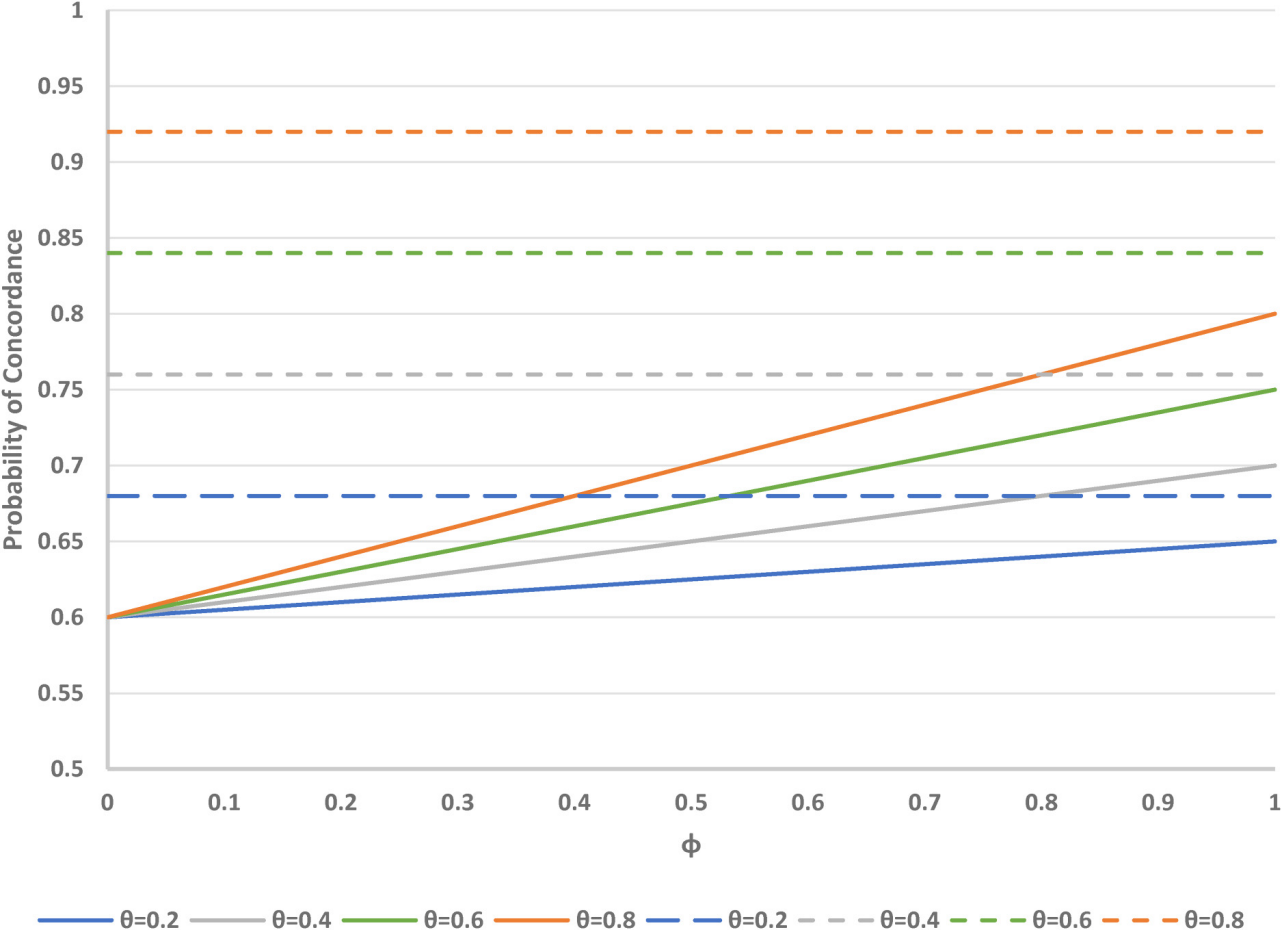
Probability of concordance versus *φ* (proportion accepting randomisation) in Zelen single consent, treatment concealed (solid lines) and treatment revealed (dashed lines) designs, for varying *θ* (proportion randomised to consent arm). Fixed parameters: *α* = 0.4 (proportion preferring treatment A); *β* = 0.15 (proportion preferring treatment B); *γ* = 0.45 (proportion undecided). *φ* = 1 is equivalent to the standard design.

Our interactive application also allows further examination of the Zelen designs compared to the standard design. If the parameters (*α*, *β*, *γ*) are regarded as fixed, the overall concordance rates in the Zelen single consent, treatments-concealed and treatments-revealed designs are shown in Figure 6 for some typical values of the preference distribution (*α* = 0.4, *β* = 0.15, *γ* = 0.45) and for the range of possible values of the randomisation parameter *θ* and the proportion *ϕ* of subjects who accept randomisation. For the treatments-concealed design, concordance increases with *ϕ*. When *ϕ* = 0, the concordance is 1 − *α* (regardless of the value of *θ*); when *ϕ* = 1, the concordance is 1 − [*α* (1 − *θ*) + *βθ*]; and when *ϕ* = *θ* = 1, maximum concordance is reached at 1 − *β*. For the Zelen single consent, treatments-revealed design, there is no dependence on *ϕ*. It is worth noting that for a given set of parameters (*α*, *β*, *γ*, *ρ*), concordance is always higher for the single consent, treatments-revealed design compared to the single consent, treatments-concealed design.

**Figure 7.**
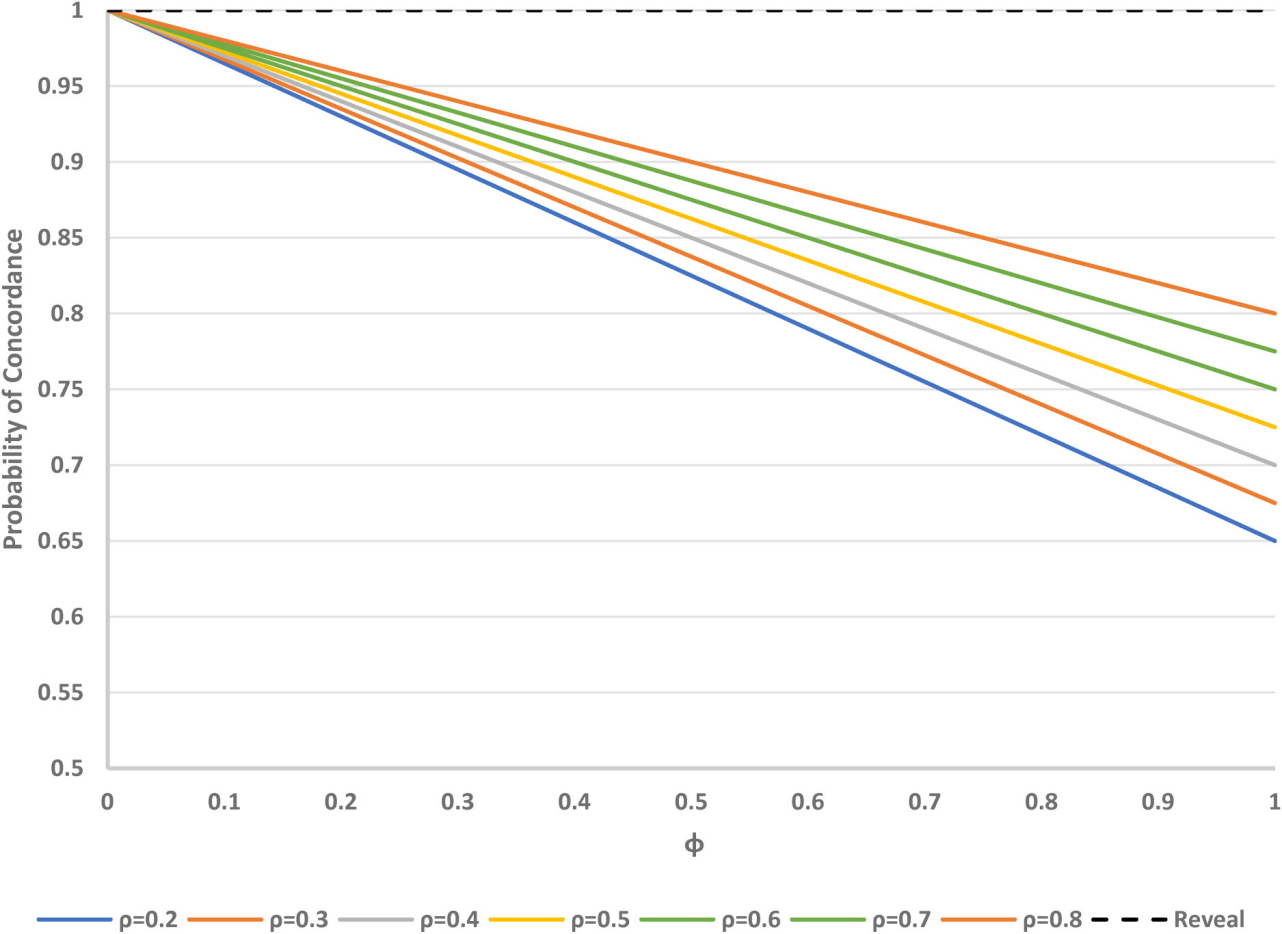
Probability of concordance versus *φ* (proportion accepting randomisation) in Zelen double consent, treatment concealed (solid lines) and treatment revealed (dashed line) designs, for varying *θ* (proportion randomised to consent arm). Fixed parameters: *α* = 0.4 (proportion preferring treatment A); *β* = 0.15 (proportion preferring treatment B); *γ* = 0.45 (proportion undecided).

As can be seen in Figure 7, if all the other parameters (*α*, *β*, *γ*, *ρ*) are regarded as fixed, the overall concordance for the Zelen double consent, treatments-concealed design decreases as *ϕ* increases. Concordance increases with *θ*. When *ϕ* = 0 (degenerate case), concordance is 1. For the Zelen double consent, treatments-revealed design, concordance is always 1, regardless of the other parameter values.

**Figure 8.**
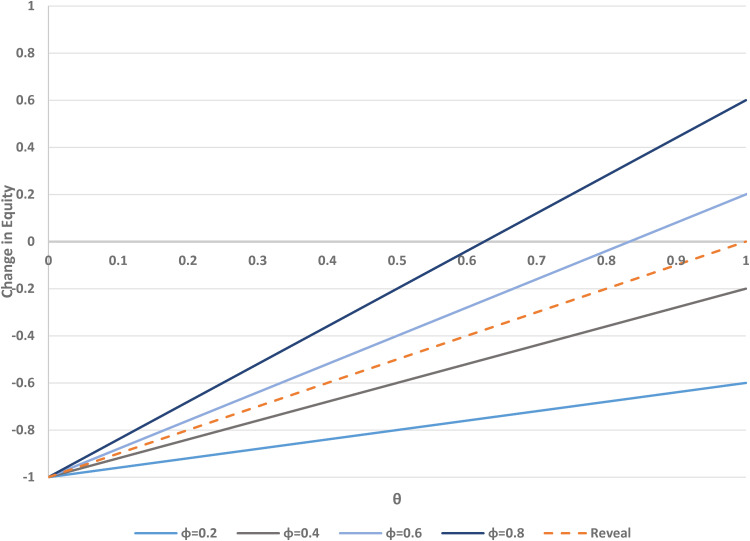
Change in equity versus *θ* (proportion randomised to consent arm) over varying *φ* (proportion accepting randomisation) in Zelen single consent, treatment concealed (solid lines) and treatment revealed (dashed line) designs. Fixed parameter: *ρ* = 0.5 (probability randomised to treatment A in standard design).

For the Zelen single consent, treatments-concealed design, there can be a large negative difference in equity compared to the standard design, as can be seen in Figure 8, for *ρ* = 0.5 (the most commonly used value). Larger changes in equity occur with larger values of *ϕ* and/or *θ*. For the single consent, treatments-revealed design and *ρ* = 0.5, the change in equity is never positive (and is not dependent on *ϕ*). Larger values of *ρ* (e.g. 0.75) result in the change in equity becoming more negative, whereas smaller values of *ρ* (e.g. 0.25) yield positive changes in equity (see Supplemental Figures 1 and 2).

**Figure 9.**
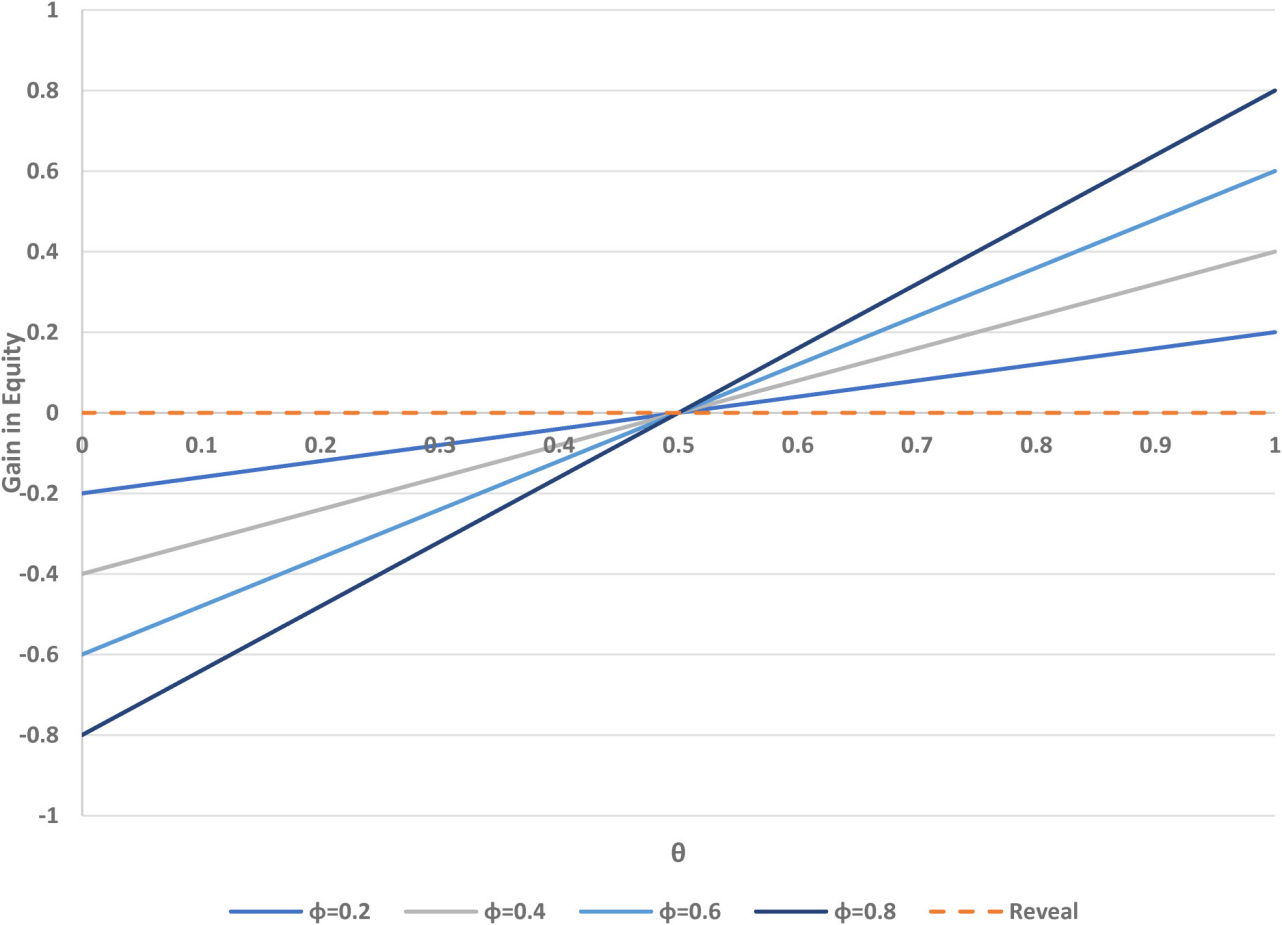
Change in equity versus *θ* (proportion randomised to consent arm) over varying *φ* (proportion accepting randomisation) in Zelen double consent, treatment concealed (solid lines) and treatment revealed (dashed line) designs. Fixed parameter: *ρ* = 0.5 (probability randomised to treatment A in standard design).

For the Zelen double consent, treatments-concealed design, there are negative changes in equity compared to the standard design when *θ* < 0.5, and positive changes when *θ* > 0.5. Holding all other parameters constant, increases in the consent rate *ϕ* translate to increases in the absolute value of the change in equity (Figure 9). For the double consent, treatments-revealed design, the equity is dependent only on *ρ*. When *ρ* = 0.5, equity is 0; when *ρ* > 0.5, equity is negative; and when *ρ* < 0.5, equity is positive. Similar to the single consent designs, changes in *ρ* result in upward (*ρ* = 0.25) or downward (*ρ* = 0.75) shifts in equity (see Supplemental Figures 3 and 4).

## 4 Illustrative example

Our illustrative example is motivated by a trial designed to determine the effectiveness of two opioid agonist medications (subcutaneous buprenorphine depot injections; sublingual buprenorphine) on the primary outcome of retention in care at 48 weeks in 18–65-year-old, opioid-dependent individuals actively seeking treatment in Australia. The original trial was designed as a two-arm, two-stage randomised, patient preference trial where estimates of preference rates and retention rates had been obtained from previous trials and feasibility studies.^16–18^

The trial setting used randomisation to the choice and random arms, and randomisation within the random arm to treatment A (subcutaneous) and treatment B (sublingual) to be 1:1 (i.e. *θ* = 0.5 and *ρ* = 0.5). The preference distribution was estimated to be (*α*, *β*, *γ*) = (0.23, 0.22, 0.55). For the Zelen design, we assume that *ϕ* = 0.86, based on the overall acceptance of opioid agonist treatment.^16^

Given these specifications, we can compare concordance and equity across the different designs. As seen in Table 5, the concordance is high for all designs, mostly because of the large proportion of individuals who are undecided. For most designs accommodating choice (except for the Zelen single consent, treatment concealed design), there is a gain in concordance over the standard parallel design.

**Table 5.** Comparison of concordance rates and equity (A vs. B) between alternative designs for an opioid agonist trial.

Design	Concordance rate	Equity	Gain in concordance
Standard parallel group	0.775	0	[Reference]
Two-stage	0.888	0	0.113
Zelen single consent, treatment concealed	0.774	−0.14	−0.001
Zelen single consent, treatment revealed	0.885	−0.50	0.110
Zelen double consent, treatment concealed	0.807	0	0.032
Zelen double consent, treatment revealed	1	0	0.225

Assumptions: all randomisations 1:1; 23% prefer treatment A; 22% prefer treatment B; 55% are undecided; 86% would accept randomisation.

## 5 Discussion

The general theory and the numerical results that we have presented show the extent to which the probability of trial participants receiving their preferred treatment is very much affected by the choice of study design. The designs also vary considerably in how well they provide an equitable experience for patients with different treatment preferences, although note that all the designs can potentially achieve the optimal equity of 0 (conditional on fixing certain parameters), so that all their patients have an equal chance of receiving their preferred treatment (see Table 2).

It is important to note that, quite apart from the personal satisfaction that patients may gain from receiving their preferred treatment, these preferences can often translate to impacts on study outcomes. In a previous extensive review of preference-based designs in practice,^1^ we considered trials with a variety of outcome types, both subjective and objective. We examined one weight loss trial in some detail, in which there were impacts of preferences on weight loss itself, and also on cholesterol, glucose, and insulin levels, all of which are objective endpoints. That review also cited many other studies with similar outcomes. Apart from this empirical evidence, we also have the general possibility that compliance with an intervention may be related to patient preferences; so, for instance, patients may be more likely to be compliant with their preferred treatment, and this in turn would again affect outcomes of all types.

Our earlier review^1^ discussed examples that more usually demonstrated improved outcomes in concordant patients (who received their preferred treatment), but counter-examples also occur (and there may be rational explanations for why this might have happened). Patient preferences may also affect whether they enrol in a trial at all, and this may or may not create a lack of external validity. Finally, as well as envisaging the effects of preferences on outcomes, we note that there may also be effects on the standard error of the estimated treatment effect.^1,9–11,14^

With a suitable design and analysis, we can get a valid (unbiased) estimate of the treatment effect, taking account of patient preferences and selection effects, wherever that is possible.^1,9–11,14^ The usual primary analyses would not allow for an interaction between the treatment effect and treatment preferences, so a change in the concordance rate would then not affect the estimated treatment benefit. On the other hand, if there is such an interaction, it could only be estimated in situations where individual patient preferences are known. However, in many cases, individual preferences remain unknown, for example in the random arm of a two-stage trial design; if so, we would then be unable to estimate treatment benefit within A-preferers or B-preferers, as would be required in order to evaluate this interaction.

As described in detail elsewhere,^1^ the two-stage design permits the study of potentially important impacts of patient preferences on study outcomes, something that is not possible with a standard design. With the two-stage design, investigators have under their control the proportion of participants that they assign to a choice arm and the randomisation rates between treatments. Using equal randomisation rates will always be advantageous in terms of guaranteeing optimal equity in either the two-stage design or the standard parallel group design. However, adopting unequal randomisation rates can potentially enhance the overall concordance rate, so that more patients receive their desired treatment. Additionally, unequal randomisation has been suggested as useful in other contexts, such as when the costs of the treatments are substantially different, where there is greater prior experience with one of the treatments, where there are concerns about adverse events or ethics, or in the face of logistical or recruitment problems.^12,13^ Careful selection of the key design parameters by the study investigators may variously optimise the overall concordance rates, the concordance rates among patient subgroups with different treatment preferences, or the equity in the design.

As mentioned above, an advantage of the two-stage design is that it can evaluate the impact of patient preferences for treatment on study outcomes in addition to the usual direct treatment effect. This can be done in an unbiased way, even though some treatments are assigned at random while others are chosen non-randomly.^1,10^ Nevertheless, one must also recognise that the direct effect of treatment will be estimated with less precision than in a standard randomised trial of the same size. When choosing their design, investigators should consider the relative importance of the preference and direct treatment effects and optimise accordingly. Optimisation involves defining which determinants of study outcomes are of greatest interest and with due attention to obtaining adequate sample sizes for each associated parameter.^1,9,11,14,19^

The fully randomised patient preference design maintains the same concordance rate and equity values as the standard design. However, there may be undesirable consequences of asking for preferences and then effectively disregarding them, at least for some patients. These consequences might include poorer compliance for patients who are not assigned their preferred treatment or increased drop-out rates from the trial. Similarly, the partially randomised patient preference design offers an ideal setting for participants in terms of universally maximising concordance and optimising equity, but it suffers from the inherent inability to estimate all relevant effects in an unbiased manner; see Gemmell and Dunn^3^ for some numerical simulations on this point.

The Zelen designs can potentially offer some gains in concordance over the standard design and may also provide closer to optimal equity. However, these designs may not be equipped to estimate selection and preference effects, and concealment of treatments at the point of obtaining consent is required to estimate the treatment effect unbiasedly; see Supplemental Appendix 1 for details. Furthermore, in our derivations for these designs, we have assumed that the probability *ϕ* of consenting to accept a randomised treatment is constant. In fact, it is possible that acceptance rates will be related to treatment preferences. For instance, based on preference, truly indifferent patients would have no rational reason to reject randomisation, whereas patients who would like the opportunity to receive an experimental treatment in a single consent Zelen design would be motivated to accept randomisation. If details of the treatments are revealed at the time of randomisation, the assumption of independence between accepting randomisation and treatment preferences becomes even less plausible. If the assumption of constant acceptance rates is not correct, valid estimation of the treatment effect becomes more problematic and complex, often to the point of impossibility.

In addition to the desirability of unbiased estimation of the effects of treatment and preferences for treatments, investigators usually also wish to complete their studies as expeditiously as possible. Therefore, they should recognise that offering an increased chance of receiving a preferred treatment will be attractive to trial participants and may therefore improve recruitment into the study.^20,21^ By this means, increasing the proportion of eligible patients who agree to participate in a trial will clearly enhance its generalisability and credibility. Furthermore, facilitation of enrolment into a trial will improve its logistical efficiency, a feature that should be borne in mind and weighed against possible losses in statistical efficiency for estimation of the direct treatment effect when preferences are taken into account by the study design. Also, in situations where patients’ preferences for treatments are strong, it is more likely that those preferences will have an impact on patient outcomes. In such cases, it should be incumbent on investigators to design their studies in such a way that these determinants of study outcomes can indeed be examined.

The work presented in this article is limited to the case when two treatments are under consideration, but our ideas could be naturally extended to include three or more treatments. We have not done so at present because the trial literature has focused largely on only two treatments.

We propose that our results can be consulted to compare concordance and equity rates for alternative designs under consideration by trial investigators. An area of future research (suggested by a reviewer) is to examine the robustness of these results to the selected values of relevant parameters. For example, one might have preliminary sample data on patient preferences for treatments, based on surveys carried out in advance of a trial; one might then assess alternative designs, taking the sample uncertainty of the preference parameters into account.

Another area for possible future research is to apply the ideas of concordance and equity to adaptive designs. In some trials, investigators may choose to modify the study design after an interim analysis, for instance by changing the randomisation ratio to assign more patients to the treatment that appears to be more beneficial at that stage. An extreme case is where study arms are eliminated for treatments that appear to have poorer outcomes. Recalling that our main focus was on patient preferences, one should note that patients should not be privy to interim results on study outcomes, and so their preference distribution should not change during the trial (for that reason, at any rate). Nevertheless, an investigator-initiated change in the design will potentially affect the overall concordance and equity rates in the later stages of a trial.

A particular type of adaptive design is where interim analyses are carried out with the potential to stop the trial early, either because there is convincing evidence of superior benefit from one of the treatments being compared, or because of futility, where it appears that a meaningful treatment is unlikely to be demonstrated, even if the trial continues to its conclusion with a full sample size. Such early stops can have an influence on the resultant estimate of the treatment effect.^22–24^ Treatment preferences by patients (and indeed, in this case, by investigators) would be unaffected before a first interim analysis. However, if there are several planned interim analyses, then, as noted above, changes in investigator preferences can occur, with potential impacts on concordance and equity in the study as a whole.

In summary, we have provided insight into some potential advantages of several trial designs that directly or indirectly recognise patient preferences for treatment, and we have documented how concordance and equity for trial participants vary between them. When choosing a study design, these factors should be considered and balanced against other criteria, including the identifiability and efficiency of estimation for various determinants of the treatment effect, possible enhancements in recruitment rates, study efficiency through patients having an improved chance of receiving their preferred treatment and increasing the fairness (or equity) in this regard for all patients in the trial.

## Supplemental Material

sj-docx-1-smm-10.1177_09622802221146305 - Supplemental material for Taking a chance: How likely am I to receive my preferred treatment in a clinical trial? Supplemental material, sj-docx-1-smm-10.1177_09622802221146305 for Taking a chance: How likely am I to receive my preferred treatment in a clinical trial? by Stephen D Walter, Ondrej Blaha and Denise Esserman in Statistical Methods in Medical Research

sj-docx-2-smm-10.1177_09622802221146305 - Supplemental material for Taking a chance: How likely am I to receive my preferred treatment in a clinical trial? Supplemental material, sj-docx-2-smm-10.1177_09622802221146305 for Taking a chance: How likely am I to receive my preferred treatment in a clinical trial? by Stephen D Walter, Ondrej Blaha and Denise Esserman in Statistical Methods in Medical Research

sj-docx-3-smm-10.1177_09622802221146305 - Supplemental material for Taking a chance: How likely am I to receive my preferred treatment in a clinical trial? Supplemental material, sj-docx-3-smm-10.1177_09622802221146305 for Taking a chance: How likely am I to receive my preferred treatment in a clinical trial? by Stephen D Walter, Ondrej Blaha and Denise Esserman in Statistical Methods in Medical Research

sj-docx-4-smm-10.1177_09622802221146305 - Supplemental material for Taking a chance: How likely am I to receive my preferred treatment in a clinical trial? Supplemental material, sj-docx-4-smm-10.1177_09622802221146305 for Taking a chance: How likely am I to receive my preferred treatment in a clinical trial? by Stephen D Walter, Ondrej Blaha and Denise Esserman in Statistical Methods in Medical Research

sj-docx-5-smm-10.1177_09622802221146305 - Supplemental material for Taking a chance: How likely am I to receive my preferred treatment in a clinical trial? Supplemental material, sj-docx-5-smm-10.1177_09622802221146305 for Taking a chance: How likely am I to receive my preferred treatment in a clinical trial? by Stephen D Walter, Ondrej Blaha and Denise Esserman in Statistical Methods in Medical Research
