# Supplementary material for: Taking a chance: How likely am I to receive my preferred treatment in a clinical trial?
Source: Stat Methods Med Res. 2023 Jan 10;32(3):572–92. doi: 10.1177/09622802221146305 (PMC9983058; doi:10.1177/09622802221146305)
Supplement: sj-docx-1-smm-10.1177_09622802221146305 - Supplemental material for Taking a chance: How likely am I to receive my preferred treatment in a clinical trial? [file sj-docx-1-smm-10.1177_09622802221146305.docx]

**Appendix: Estimability of the treatment, selection, and preference effects in the Zelen single- and double-consent trial designs.**

Here we describe which parameters of interest can be estimated in the various Zelen designs, the formats of which are shown in Figures 1(e) and 1(f). We adopt a linear model for the various effects on a continuous outcome variable *Y*:

*Y_ijk_ = μ + τ_i_ + υ_j_ +* *π_ij_ + ε_ijk_* (1)

where *$Y_{ijk}$* is the outcome for patient *k* receiving treatment *i* (*i* = 1 for treatment A, 2 for treatment B) and preferring treatment *j* (*j* = 1 for participants preferring A, *j* = 2 for participants preferring B, and *j* = 3 for participants with no preference). The parameters *τ*, *ν*, and *π* represent the treatment, selection, and preference effects, as defined below; *ε* is a random error term, assumed to be independent of the other terms, and having mean zero.

We apply four sets of constraints to the parameters in order to avoid redundancy because of over-parameterisation, as follows:

*$\tau_{1}+\tau_{2}=0$,*

*$\alpha\nu_{1}+\beta\nu_{2}+\gamma\nu_{3}=0$,*

*$\alpha\pi_{i1}+\beta\pi_{i2}+\gamma\pi_{i3}=0$,* for *i* = 1, 2

*$\pi_{1j}+\pi_{2j}=0$* for *j* = 1, 2, 3,

where *α,* *β,* and *γ* define the preference distribution (with *$\alpha+\beta+\gamma=1$*), giving the expected proportions of participants who prefer treatment A, treatment B, or are indifferent or undecided, respectively. Because *α, β,* and *γ* are unknown parameters, the second and third constraints on the *υ* and *π* terms are variable.

Three contrasts are of potential interest:

a) The Treatment Effect (denoted by *TE*), which is usual direct effect of treatment. In terms of the parameters in (1), *TE* ***=*** *τ****_1_ –*** *τ****_2_***.

b) The Selection Effect (*SE*), defined as the expected difference in outcomes between patients who would choose A (if allowed to do so) and those who would choose B (if allowed to do so). In the model (1) notation, *SE* ***=*** *ν_1_ – ν_2_****.***

c) The Preference Effect (*PE*) is defined as *PE* ***=*** *π_11_ – π_12_ – π_21_ + π_22_****.*** This is equivalent to the interaction between actual and selected treatments, and it reflects the difference in treatment effects between participants who do or do not receive their preferred treatment.

For other designs, we previously also previously [1] described the Concordance effect (*CE*), which represents the difference between outcomes on treatments A vs. B for those who receive their selected treatment and those who do not. In the model (1) notation, *CE = (π_11_ – π_22_) – (π_12_ - π_21_),* but this quantity is not identifiable in any of the Zelen designs.

We now consider the Zelen single- and double-consent trial designs, whose structure is shown in Figure 1. In both cases, details of the experimental treatment may be either concealed or revealed at the time of seeking consent. For each of the resultant four designs, we now derive the expected outcomes in each of their study groups, and hence establish which of the contrasts above can be estimated.

*Single consent, treatment-concealed design*

Referring to model (1), if we assume that the probability of giving consent to receiving treatment A is independent of treatment preferences, then the preference distribution among patients who are randomised to the consent arm and accept treatment A is the same as in the patients at large. Hence the expected outcome in this group, averaged over the treatment preference distribution, is

$$\mu_{1} = \mu+\tau_{1} +\alpha\left( \upsilon_{1} + \pi_{11} \right)+ \beta\left( \upsilon_{2} + \pi_{12} \right)+ \gamma(\upsilon_{3} + \pi_{13})$$

Recalling the parameter constraints, this reduces to:

Randomised to consent arm and give consent (receive treatment A): $\mu_{1} = \mu+\tau_{1}$

Similarly, the expected outcomes in the other two study groups are:

Randomised to consent arm and refuse consent (receive treatment B):

$\mu_{2} = \mu+\tau_{2}$

Randomised to control arm (receive treatment B):

$\mu_{3} = \mu+\tau_{2}$

Collectively, these outcomes show that the contrast between the first group and the other two groups (singly or combined) provides an unbiased estimate of the treatment effect TE. However, these are the only available contrasts, and hence SE and PE are not identifiable. Furthermore, if there is actually a dependence between the probability of giving consent and treatment preferences (for instance, if patients who are strongly in favour of getting a new treatment are more likely to grant consent), then SE and PE remain unidentifiable, and the estimate of TE becomes potentially biased by inherent selection and preference effects.

*Single consent, treatment-revealed design*

In this design, details of the experimental treatment A are revealed at the stage of requesting consent in the consent arm. Patients who are randomly assigned to the control treatment B are not asked for consent. We assume that undecided patients accept treatment A if they are randomised to the consent arm. The expected outcomes in the three study groups are therefore:

Randomised to consent arm and accept treatment (receive treatment A):

$\mu_{1} = \mu+\tau_{1}+\left[ \alpha\left( \upsilon_{1}+\pi_{11} \right)+\gamma\left( \upsilon_{3}+\pi_{13} \right) \right]/(\alpha+\gamma)$

Randomised to consent arm and refuse treatment (receive treatment B):

$\mu_{2} = \mu+\tau_{2}+ \upsilon_{2}+\pi_{22}$

Randomised to control arm (receive treatment B):

$\mu_{3} = \mu+\tau_{2}$

Contrasts between the first group and the others necessarily involve selection and preference parameters, and hence estimates of TE are potentially biased by these effects. SE and PE are again not identifiable.

*Double consent, treatment-concealed design*

We continue to assume that giving consent is independent of treatment preferences, and that undecided patients give consent to receiving either treatment, if asked. Numbering the patient outcome means in the patient groups in Figure 1 from left to right, the expected outcomes among patient who grant consent are then:

Randomised to A-consent arm, accept, receive treatment A: $\mu_{1} = \mu+\tau_{1}$

Randomised to B-consent arm, accept, receive treatment B: $\mu_{3} = \mu+\tau_{2}$

Comparison of these two groups provides an unbiased estimate of the treatment effect. Patients who refuse consent receive their preferred treatment; conditional on the treatment received, their expected outcomes are the same regardless of whether they had been asked to consent to A or B. With an obvious notation, their expected outcomes are therefore:

Refuse consent, receive treatment A: $\mu_{2A}= \mu_{4A}= \mu+ \tau_{1}+ \upsilon_{1}+\pi_{11}$

Refuse consent, receive treatment B: $\mu_{2B}= \mu_{4B}= \mu+ \tau_{2}+ \upsilon_{2}+\pi_{22}$

In the special case where there are no undecided patients ($\gamma=0)$, the set of four study groups (randomised with consent to A or B; refuse consent and receive A or B) is equivalent to a two-stage randomised design, and hence valid estimates of SE and PE can be obtained [1, 8, 9]. In an alternative scenario where undecided patients in the consent arms are randomised to treatments, once again the three effects of interest (TE, SE, and PE) can still be estimated [10, 11].

*Double consent, treatment-revealed design*

In this design, details of treatments A or B are revealed when consent is sought for either of the treatments. Patients who refuse to consent receive their preferred treatment. Again assuming that indifferent patients are assigned to the particular treatment for which their consent is requested, the expected outcomes in the four patient groups are now:

Randomised to A-consent arm, accept, receive treatment A:

$\mu_{1} = \mu+\tau_{1}+\left[ \alpha\left( \upsilon_{1}+\pi_{11} \right)+\gamma\left( \upsilon_{3}+\pi_{13} \right) \right]/(\alpha+\gamma)$

Randomised to A-consent arm, refuse, receive treatment B:

$\mu_{2} = \mu+\tau_{2}+ \upsilon_{2}+\pi_{22}$

Randomised to B-consent arm, accept, receive treatment B:

$\mu_{3} = \mu+\tau_{2}+\left[ \beta\left( \upsilon_{2}+\pi_{22} \right)+\gamma\left( \upsilon_{3}+\pi_{23} \right) \right]/(\beta+\gamma)$

Randomised to B-consent arm, refuse, receive treatment A:

$\mu_{4} =\mu+\tau_{1}+ \upsilon_{1}+\pi_{11}$.

This design provides no randomised comparisons of the treatments, hence no unbiased estimate of TE is available, and SE and PE are not identifiable.
