# Supplementary material for: Taking a chance: How likely am I to receive my preferred treatment in a clinical trial?
Source: Stat Methods Med Res. 2023 Jan 10;32(3):572–92. doi: 10.1177/09622802221146305 (PMC9983058; doi:10.1177/09622802221146305)
Supplement: sj-docx-4-smm-10.1177_09622802221146305 - Supplemental material for Taking a chance: How likely am I to receive my preferred treatment in a clinical trial? [file sj-docx-4-smm-10.1177_09622802221146305.docx]

Supplemental Figure 3: Change in equity vs. *θ* (proportion randomized to consent arm) over varying φ (proportion accepting randomization) in Zelen double consent, treatment concealed (solid lines) and treatment revealed (dashed line) designs. Fixed parameters: *ρ* = 0.25 (probability randomized to treatment A in standard design).
